# Autologous Fat Transplantation for Thumb Carpometacarpal Joint Osteoarthritis (Liparthroplasty): A Case Series with Two Years of Follow-UP

**DOI:** 10.3390/jcm10010113

**Published:** 2020-12-31

**Authors:** Stefan M. Froschauer, Matthias Holzbauer, Raphael Wenny, Manfred Schmidt, Georg M. Huemer, Oskar Kwasny, Dominik Duscher

**Affiliations:** 1 Department for Trauma Surgery and Sport Traumatology, Med Campus III, Kepler University Hospital Linz, Krankenhausstrasse 3, 4020 Linz, Austria; matthias.holzbauer@a1.net (M.H.); Oskar.kwasny@kepleruniklinikum.at (O.K.); 2 Faculty of Medicine, Johannes Kepler University Linz, Altenbergerstraße 69, 4020 Linz, Austria; manfred.schmidt@kepleruniklinikum.at; 3 Department of Plastic, Aesthetic, and Reconstructive Surgery, Med Campus III, Kepler University Hospital Linz, Krankenhausstrasse 3, 4020 Linz, Austria; raphael.wenny@kepleruniklinikum.at (R.W.); georg.huemer@kepleruniklinikum.at (G.M.H.); dominikduscher@me.com (D.D.); 4 Department of Plastic, Reconstructive, Hand and Burn Surgery, BG-Trauma Center, Eberhard Karls University Tuebingen, Schnarrenbergstrasse 95, 72076 Tuebingen, Germany

**Keywords:** arthroplasty, autologous fat grafting, liparthroplasty, thumb carpometacarpal osteoarthritis

## Abstract

Adipose-derived mesenchymal stem cell (ASC) therapy is currently a focus of regenerative medicine. Lipoaspirate is rich in ASCs and is evolving into a promising, less-invasive tool to treat thumb carpometacarpal osteoarthritis as compared with common surgical techniques, for example, trapeziectomy or prosthesis implantation. The present study aimed to examine the effect of 1 mL intraarticular lipoaspirate injection (liparthroplasty) in 31 thumb carpometacarpal osteoarthritis patients (27 woman and four men) with a median age of 58 (interquartile range (IQR) of 10) years and Eaton–Littler Stage 2 or 3. Median pain levels assessed via visual analogue scale significantly decreased from 7 (IQR 2) to 4 (IQR 6) after six months (*p* < 0.0001) and 2 (IQR 5) after two years (*p* < 0.0001). Median pre-interventional Disabilities of the Arm, Shoulder and Hand (DASH) scores of 59 (IQR 26) significantly reduced to a value of 40 (IQR 43) after six months (*p* = 0.004) and to 35 (IQR 34) after two years (*p* < 0.0001). Subjective grip strength showed no significant improvement. However, the time until recurrence of symptoms was measured and a cumulative remission rate of 58% was detected after two years. Satisfaction rates were 68% after six months and 51% after two years. In conclusion, liparthroplasty represents a promising option to reduce pain and functional impairment and to postpone surgery for a certain period of time.

## 1. Introduction

Thumb carpometacarpal (CMC) joint osteoarthritis (OA) is a disabling condition especially affecting elderly, postmenopausal woman [1]. In early OA stages, the therapeutic approach starts with conservative methods, for example, physiotherapy, orthoses, or systemic antirheumatic drugs [2]. If these options do not yield the desired success, therapy can be escalated in a further step by injecting corticosteroid into the thumb CMC joint. Despite correct intraarticular injection, extravasation has been found in 33% of cases, which leads to atrophy of fat tissue, structural impairment of tendons and capsular ligaments, as well as hyperpigmentation of the skin [3]. In addition to corticosteroids, injection-based treatment using hyaluronic acid, saline placebo, and dextrose were compared in a recent meta-analysis which concluded that there was no clear evidence whether any injected substance was superior to others or that one of them was better than another conservative treatment option [4]. As a further consequence, if non-operative treatment fails, a wide range of different surgical methods are available, for example, denervation, partial or total trapeziectomy, resections-suspension arthroplasty with use of various adjacent tendons, arthrodesis, and total arthroplasty with a huge number of various prosthesis types [5,6,7].

Because, so far, conservative treatment options only result in temporary, short-term pain relief, autologous lipoaspirated fat transplantation (AFT) to the thumb CMC joint (liparthroplasty) represents a promising approach to both reduce pain for a long period, and hence to postpone the necessity for thumb CMC surgery, as well as to preserve the bone stock of the trapezium and first metacarpal as long as possible.

Lipoaspirate has been shown to be rich in adipose-derived mesenchymal stem cells (ASC). Recently, several studies have focused on the regenerative effect of mesenchymal stem cells [8,9,10]. Basic research conducted on mice suffering from experimental collagenase-induced OA revealed an immunosuppressive effect of ASCs, which inhibited synovial thickening and cartilage destruction [9]. Beneficial effects of AFT have already been detected in clinical trials conducted in various joints affected by OA, for example, femoropatellar joint [8], knee [11,12,13,14], hip [11], ankle [11], and thumb CMC joint [15,16,17,18]. The safety of injecting ASC into human joints has already been proven in a study with a five-year follow-up period, in which no neoplastic complications could be detected [11]. According to a recent review [19], AFT into the thumb CMC joint has been previously examined in studies with a maximum follow-up period of 18 months [20]. The main effects of this therapy were found to be reduced pain and functional impairment related to this degenerating condition, which were assessed using either the Disabilities of the Arm, Shoulder and Hand (DASH) score or the Michigan Hand Questionnaire. Moreover, by comparing AFT to already established common therapies, it proved to show advantages as compared with corticosteroid injection regarding the previously mentioned parameters and revealed comparable results to Lundborg resection-suspension arthroplasty [15,20].

The hypotheses that try to explain the therapy’s success in thumb CMC joint range from a simple placebo to a cushioning effect, or even chondroregenerative effects [15,20,21].

On the basis of the previously published, promising data of AFT, the present study aims at examining the clinical outcomes of lipoaspirate harvested from the abdominal region injected into arthritically degenerated thumb CMC joints over a systematic follow-up period of two years.

## 2. Experimental Section

Between February 2017 and August 2018, we enrolled both female and male patients with painful and radiographically confirmed thumb CMC joint OA. Eaton–Littler Stage 2 to 3 and exhausted conservative treatment, i.e., a stabilizing thumb orthosis, antiphlogistic drugs, and hand therapy, were defined as inclusion criteria. We excluded patients with any history of trauma (e.g., Bennett’s fracture-dislocation or Rolando fracture), rheumatoid arthritis, concomitant scapho-trapezio-trapezoid OA, or any previous surgery of the thumb CMC joint.

This study was approved by the local ethical review board (EK no. 1094/2018) and was conducted according to the World Medical Association (WMA) Declaration of Helsinki. All patients gave their written informed consent to participate in this trial.

### 2.1. Liparthroplasty Technique

First, lipoaspirate was harvested in regional anesthesia from the abdominal region using a single umbilical incision. After infiltrating 50 milliliters of tumescent solution (1000 mL of saline solution, 1 mL of 1:200,000 adrenalin, and 600 mg of lidocaine), fat was obtained from the lower abdomen. The fat tissue was separated from fluids and oils using the decanting method without the need for being centrifuged. Next, the fat was mechanically homogenized with two syringes (shuffling method), which were connected to a three-way valve. A single-use 20-gauche needle with a 3 mL syringe was used to inject 1 mL of lipoaspirate into the thumb CMC joint under fluoroscopic guidance. Wound closure was performed using a sterile, self-adhesive dressing. Moreover, a stabilizing thumb orthosis was applied for 3 weeks post intervention, while we allowed exercising up to the individual pain threshold. Postoperative therapeutic regimen did not include hand therapy.

Every lipoaspirate injection into the thumb CMC joint was performed by a single Level 5 surgeon, while liposuctions were obtained by 3 different surgeons, which consisted of two Level 4 surgeons and one Level 2 surgeon [22].

### 2.2. Clinical Assessment

Prior to the intervention, baseline DASH scores and pain levels using the visual analogue scale (VAS), as well as the patient’s body mass index (BMI) were assessed. After 4, 8, and 16 weeks, patients were examined on how symptoms had changed as compared with the preoperative condition, using a Likert scale with 5 items as follows: 1 equaled “much better”, 2 represented “better”, 3 meant an “unaltered status”, whereas 4 was “worse”, and 5 was “much worse”. Six months post-intervention, we assessed DASH Scores, pain via VAS, and subjective grip parameter using the same Likert Scale as described above, whereas the patients were asked to describe the current grip force as compared with the preoperative one. The final follow-up appointment was conducted 2 years after the intervention, where we assessed the DASH score, the VAS level, the subjective grip, as well as the pinch strength using a Jamar dynamometer.

The DASH score reduction was determined as the primary outcome endpoint. Data of patients, who were not able to finish our postinterventional follow-up regimen, were analyzed when data were available.

Moreover, any complications and any potential time until recurrence of initial symptoms were noted. All these assessments were conducted by a single observer.

### 2.3. Statistical Methods

The sample size calculation was based on the primary outcome variable, i.e., the DASH score. Regarding this questionnaire, Sorensen et al. reported a minimal clinically important difference of 10 points [23]. Thus, the null hypothesis was that liparthroplasty yields a DASH score reduction of <10 points. Considering a Wilcoxon test with non-normal data distribution, an α of 0.05%, power of 80%, a difference in means of 10 points, and a standard deviation of 16, which was published by Ehrl et al. for a comparable study cohort consisting of Stage 2 and 3 OA patients, a minimum of *n* = 26 patients was required for this study [24]. Assuming a loss of follow-up rate of 20% in our per protocol analysis, 31 patients were necessary.

A descriptive analysis of demographic and clinical data was performed. The Kolmogorov–Smirnov test was applied to examine if each outcome parameter was normally distributed. Outcome data were either presented using a box plot, as mean and standard deviation (SD) for normally distributed parameters or as median and interquartile range (IQR) in the case of non-normal data. A Kaplan–Meier plot was created to visualize the cumulative remission function.

A Friedman test was performed to compare subjective clinical improvement DASH scores and VAS assessments. If this test revealed a significant difference between time of measurements, Dunn–Bonferroni post hoc tests were carried out. Adjusted significant values were reported. Concerning pain measurement, a Wilcoxon test was carried out. Moreover, satisfaction rates were compared using a Pearson’s Chi-squared test. A *p*-value < 0.05 was considered to be significant.

A subgroup analysis was performed to detect any differences between Eaton–Littler Stage 2 and Stage 3 OA. The unpaired *t*-test and the Mann–Whitney U test were used to compare the following outcome variables: age, DASH scores, pain via VAS, Likert scale concerning symptom’s change, subjective, and objective grip strength. Moreover, the Fisher’s exact test was used to test differences in proportions; hence, this test was used to compare sex, the affected side, and satisfaction rates. Moreover, we performed a post-hoc power analysis for this subgroup analysis.

## 3. Results

### 3.1. Patients’ Demographics

Initially, we enrolled 27 women and four men in the present study, of which eight women could not attend our final follow-up appointment because their treatment regimen had to be escalated to a more invasive, joint replacing surgery after six months. The median age at time of surgery was 57.7 (IQR 10.0) years with a range of 46.4 to 78.3 years. We assessed 13 left and 18 right thumbs, of which seven thumbs revealed Stage 2 osteoarthritis and 24 thumbs revealed Stage 3 osteoarthritis. The median BMI was 26.6 (IQR 7.2).

### 3.2. Subjective and Functional Parameters

The Likert scale concerning clinical improvement revealed a median result of 3 (IQR 1) at four weeks, while we could detect a value of 2 (IQ 1) at eight weeks and 2 (IQR 2) at 16 weeks. Although the Friedman test revealed a significant result (*p* = 0.002), there was no significant result detectable in the pairwise comparisons after applying the Bonferroni correction for multiple tests.

**Figure 1:**
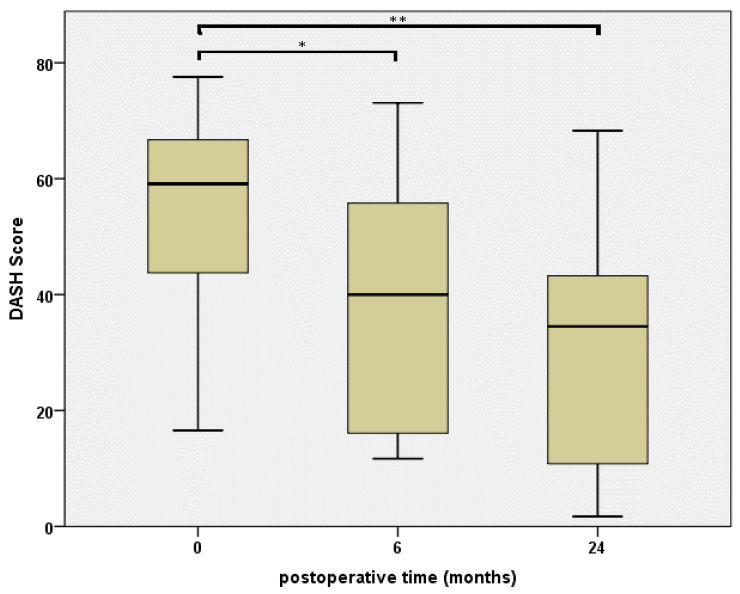
Disabilities of the Arm, Shoulder and Hand (DASH) score results prior the intervention, as well as 6 and 24 months postoperatively, illustrated in box plots. Statistical comparison is indicated using * for a *p*-value of 0.004 and ** for a *p*-value of <0.0001.

DASH scores measured preoperatively, as well as six months and two years after surgery, are displayed in Figure 1. After performing the Friedman test (*p* < 0.0001), the Bonferroni correction revealed a significant result for two years vs. preoperative values (*p* < 0.0001) as well as six months vs. preoperative values (*p* = 0.004). No significancy could be detected between the two postoperative results (*p* = 0.17).

**Figure 2:**
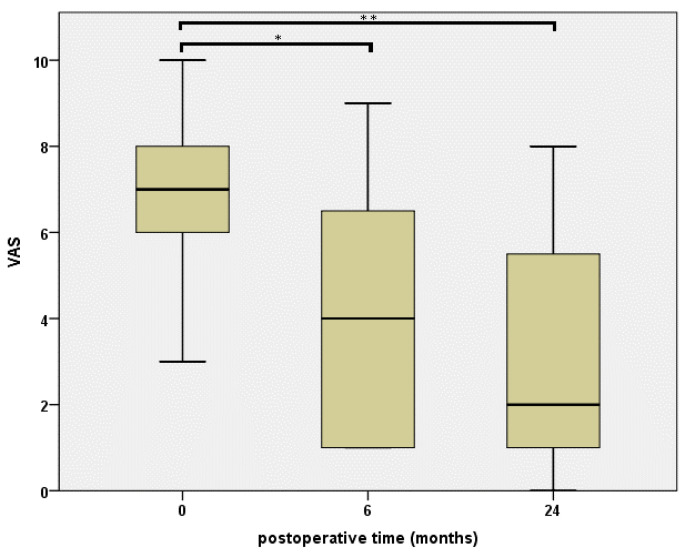
Pain levels via visual analogue scale (VAS) at a baseline, as well as 6 and 24 months after the intervention. *P*-values resulting from statistical testing are displayed using * for a *p*-value of <0.0001 and ** for a *p*-value of <0.0001.

Concerning our pain measurement using the VAS, we could detect a highly significant difference (*p* < 0.0001) comparing two years vs. preoperative and six months vs. preoperative results, which are illustrated in Figure 2. Again, in the course of follow-up, no significant reduction could be detected comparing two years and six months results (*p* = 1.0).

Comparing the subjective grip strength, the results measured at six months were 0 (IQR 0) and at two years 0 (IQR 1). While comparing these results, no significant result could be detected (*p* = 1.0). Moreover, the dynamometer pinch strength measurement at our final follow-up appointment showed a median grip strength of 6.5 (IQR 2.5) kilogram.

**Figure 3:**
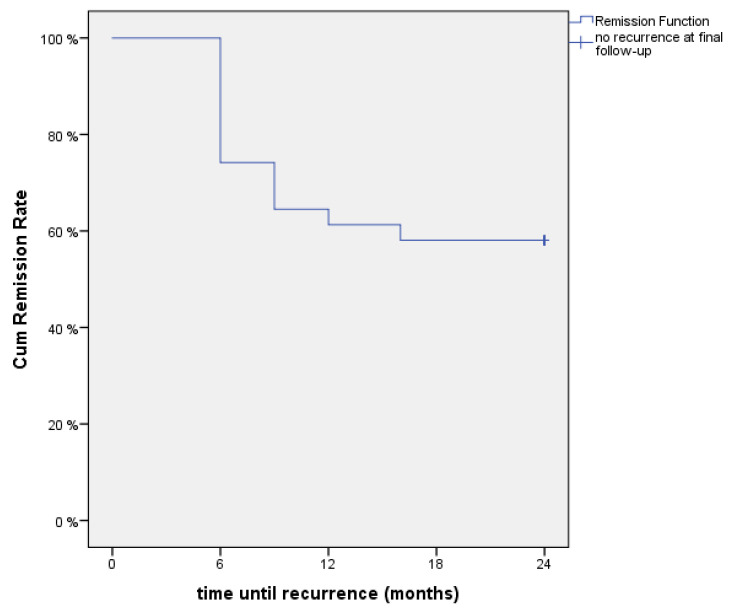
The time until recurrence of symptoms is displayed using a Kaplan–Meier plot resulting in a cumulative remission rate function.

At six months postintervention, 21 of 31 patients (68%) were satisfied with the procedure, while two years after the liparthroplasty, 16 of 31 patients (51%) were content with the result. Comparing these results, we could not detect a significant difference (*p* = 0.196). Furthermore, the time until recurrence of initial, pre-interventional symptoms resulting in a cumulative remission rate function is displayed in Figure 3, using a Kaplan–Meier plot.

Moreover, no postoperative complications were noted, for example, infection, sign of adverse reactions, or side effects.

### 3.3. Subgroup Analysis Regarding Radiographic Stage

The subgroup analysis on the basis of the radiographical stage in Table 1 shows descriptive statistics of Eaton–Littler Stage 2 and 3 patients, as well as a statistical comparison between the outcome values including statistical tests and *p*-values.

According to our primary outcome variable, the DASH score, a post-hoc power of 0.12 and 0.05 was calculated for the measurement after six months and two years, respectively.

## 4. Discussion

The results of the present study show that AFT to CMC I joints (liparthroplasty) is a valuable therapy option for thumb CMC joint OA, especially in terms of reducing pain and functional impairment over a two-year follow-up period. Previously published studies have reported comparable favorable effects of simple AFT to the thumb CMC joint, up to a maximum follow-up period of 18 months [15,16,17,18,19,20].

The first pilot study concerning this novel treatment approach was conducted by Herold et al., in 2014, with a series of five patients [17]. They were able to report a reduction of pain values under stress from 7.4 to 2.4 within three months. Three years later, the same study group published their results of 50 patients with a follow-up period of one year and validated their initial findings by observing a significant pain reduction from 7.7 to 2.4 concerning Stage 2 OA patients, while lower pain-relieving effects for Stages 3 and 4 OA were reported [18]. Moreover, Hass et al. published a constant, linear reduction of pain levels in their recent case review of 99 joints, starting with a preoperative VAS value of 6.6 downwards to 3.7 at the final follow-up appointment one year postoperatively [15]. These findings are in accordance with our presented results. We are also able to show a continuous postoperative decrease in median VAS values up to 2.0 after two years with comparable baseline values.

According to a previously published report, Erne et al. presented a similar VAS level of 2.9 in their case-control study with the currently longest mean follow-up period (18.1 months) for simple AFT [20]. This unique study comparing Lundborg resection-suspension arthroplasty with AFT, in which both pursued the common purpose of pain reduction, revealed a satisfactory postoperative decline in pain levels for both procedures [20]. Moreover, the authors concluded that fat injection led to a significantly shorter time until the absence of pain as compared with surgical intervention. In contrast to this evaluation regarding resolution of symptoms, we assessed the time until recurrence of the initial symptoms. Accordingly, the Kaplan–Meier plot (Figure 3) demonstrated that 25% of patients did not benefit from the procedure after six months. Subsequently, however, the cumulative remission rate became flatter, hence, this novel technique seemed to yield a constant, favorable therapeutic effect or remission of symptoms up to 24 months in 58% patients.

Moreover, Haas et al. reported that AFT proved to be superior regarding pain at rest, pain under stress, and DASH scores as compared with intraarticular cortisone injection [16].

Concerning the functional outcome evaluation, several authors have reported a significant postoperative reduction of DASH scores. Herold et al. published a mean DASH score of 33 after three months, as well as a significant postoperative decreased score for their overall study population after 12 months [17]. Erne et al., who refrained from including baseline data in their publication, described a mean value of 24 after 18 months, which was not significantly different from the mean value of the Lundborg cohort (22 after on average 23.6 months) [20]. Our results are in accordance with this range of postoperative values. Additionally, we corroborated the findings of Haas et al. that a continuous improvement of functional scores over the course of follow-up could be observed, even though those authors applied the Michigan Hand Outcomes Questionnaire for their study. In contrast, however, Herold et al. described an initial strong reduction of DASH values after six months, which subsequently increased again up to their final follow-up period of 12 months [18].

In addition to pain and functional improvement, liparthroplasty reaches its limits in terms of other clinical parameters. Neither the present study nor most previous publications, except for Herold et al. [18], detected a significant increase in grip or pinch strength, respectively [15,17,20]. Our final follow-up results show similar absolute grip strength values. Moreover, Erne et al. could not detect any difference concerning the OA stage while comparing the preoperative X-rays to the X-rays taken one year postoperatively [20]. Furthermore, Haas et al. discontinued assessing the range of motion and the Kapandji score during their data collection because no change could be detected [15].

In contrast to the liparthroplasty method applied in the present study, the literature revealed surgical techniques combining AFT with an external distractor for two weeks applied to preserve radiologic joint space [25], as well as with arthroscopic synovectomy, which led to considerable pain and QuickDASH score reduction [26]. Moreover, Bohr et al. published a case report regarding an injection therapy with stromal vascular fraction, which represented lipoaspirate enriched with a potentially regenerative mesenchymal cell suspension [21]. The hypothesis underlying this extra step was to maximize the concentration of ASC in the graft to yield the best possible effect of prolonged secretion of different growth factors acting chondroprotective or chondroregenerative. Theoretically, tissue-specific differentiation with subsequent synovial integration of the transplanted ASCs has been hypothesized [21]. However, the synovial space represents a relatively harsh environment for grafted cells to survive, thus, no evidence for viable fat using magnetic resonance imaging three months postoperatively, or radiologically observed changes after one year, could be detected [20,25]. Therefore, we hypothesize that the therapeutic effect of liparthroplasty, in our study, is caused by a combination of an anti-inflammatory effect and its mechanical function. The transplanted fat tissue might serve as a buffer cushioning the forces acting between both articulation joint surfaces during movement. Moreover, it probably acts as a lubricant, which might have superior sliding properties for arthritically degenerated cartilage as compared with physiological synovia.

The present study also has some limitations. In addition to the pinch strength evaluation at our final follow-up appointment, we included a subjective grip strength parameter in our assessment. We acknowledge that it may be difficult for study participants to estimate present grip strength as compared with that six months or two years ago. Due to the necessity of a treatment change in eight patients, our outcome data acquisition after two years was conducted on 74% of initial subjects. We showed, to the best of our knowledge, the longest follow-up period for AFT to the thumb CMC joint in the literature. However, further studies in a randomized, control trail design with larger cohorts and longer follow-up periods are required to evaluate the reliability, as well as the duration of effectiveness, of this promising method. Although we could detect significantly lower pain levels in Stage 2 OA patients as compared with the patients suffering from Stage 3 OA after two years, our subgroup analysis implies a low statistical power due to a small number of patients. Thus, further investigations would be beneficial to detect the ideal radiographic or clinical OA stage when liparthroplasty should be performed to obtain the most favorable results. Furthermore, it still needs to be determined if our findings concerning the liparthroplasty method could also be transferred to other human joints that are affected by an arthritic condition.

## 5. Conclusions

The outcomes of our present study show that AFT into the thumb CMC joint (liparthroplasty) provides significant relief of pain, as well as improvement of functional scores for patients, who suffer from Stage 2 and 3 OA. However, it does not generally replace the necessity for thumb CMC surgery, because liparthroplasty does not impact other clinical parameters and implicates a limited duration of effectiveness in more than one-third of patients after two years, respectively. Thus, this intervention seems to be a valuable, symptomatic bridging therapy, which preserves bone stock and postpones common, more invasive procedures as long as possible. However, further studies are required to investigate the duration of effectiveness, the underlying treatment effects, as well as to define the method’s role in the treatment regimen of the thumb CMC joint OA.

## Figures and Tables

**Table 1:** Subgroup analysis comparing Stage 2 to Stage 3 osteoarthritis (OA) outcome data. Statistically significant values are printed in bold.

Parameter	Time	Scale	Stage 2	Stage 3	Test	*p*-Value
Incl. patients	Preop.	No.	7	24	-	-
Age in years	Preop.	median (IQR)	57.3 (10.3)	57.7 (13.9)	Mann–Whitney U test	0.449
Sex	-	female/male	7/0	20/4	Fisher’s exact test	0.550
Side	-	left/right	2/5	11/13	Fisher’s exact test	0.667
DASH score	Preop.	median (IQR)	61 (28)	59 (27)	Mann–Whitney U test	0.532
Pain via VAS	Preop.	mean (SD)	6.6 (2.7)	6.8 (2.0)	Unpaired *t*-test	0.778
Symptoms Likert	4 Week	median (IQR)	3 (1)	3 (1)	Mann–Whitney U test	0.980
Symptoms Likert	8 Week	median (IQR)	2 (1)	2 (1)	Mann–Whitney U test	0.917
symptoms Likert	16 Week	median (IQR)	2 (1)	2 (1)	Mann–Whitney U test	0.053
DASH score	6 Month	mean (SD)	33 (17)	40 (22)	Unpaired *t*-test	0.456
Pain via VAS	6 Month	median (IQR)	1.0 (3.0)	4.0 (5.6)	Mann–Whitney U-test	0.052
Subj. grip	6 Month	median (IQR)	0 (1)	0 (0)	Mann–Whitney-U-test	0.229
Satisfaction	6 Month	no/yes	0/7	10/14	Fisher’s exact test	0.066
DASH score	2 Year	median (IQR)	31 (32)	38 (34)	Mann–Whitney U test	0.658
Pain via VAS	2 Year	mean (SD)	1.3 (1.0)	3.6 (2.9)	Unpaired *t*-test	0.010
Subj. grip	2 Year	median (IQR)	1 (1)	0 (0)	Mann–Whitney U test	0.201
Obj. grip in kg	2 Year	mean (SD)	6.3 (1.5)	6.4 (1.8)	Unpaired *t*-test	0.819
Satisfaction	2 Year	no/yes	1/6	14/10	Fisher’s exact test	0.083
Treatment change	2 Year	no/yes	6/1	17/7	Fisher’s exact test	0.642

## Data Availability

Data is contained within the article or supplementary material.
